# *In vivo* functional expression of a screened *P. aeruginosa* chaperone-dependent lipase in *E. coli*

**DOI:** 10.1186/1472-6750-12-58

**Published:** 2012-09-06

**Authors:** Xiangping Wu, Pengyong You, Erzheng Su, Jingjing Xu, Bei Gao, Dongzhi Wei

**Affiliations:** 1 State Key Laboratory of Bioreactor Engineering, New World Institute of Biotechnology, East China University of Science and Technology, Shanghai, 200237, China

**Keywords:** *Pseudomonas aeruginosa*, Lipase, Chaperone, Dual expression plasmid, Esterification

## Abstract

**Background:**

Microbial lipases particularly *Pseudomonas* lipases are widely used for biotechnological applications. It is a meaningful work to design experiments to obtain high-level active lipase. There is a limiting factor for functional overexpression of the *Pseudomonas* lipase that a chaperone is necessary for effective folding. As previously reported, several methods had been used to resolve the problem. In this work, the lipase (LipA) and its chaperone (LipB) from a screened strain named AB which belongs to *Pseudomonas aeruginosa* were overexpressed in *E. coli* with two dual expression plasmid systems to enhance the production of the active lipase LipA without *in vitro* refolding process.

**Results:**

In this work, we screened a lipase-produced strain named AB through the screening procedure, which was identified as *P. aeruginosa* on the basis of 16S rDNA. Genomic DNA obtained from the strain was used to isolate the gene *lipA* (936 bp) and lipase specific foldase gene *lipB* (1023 bp). One single expression plasmid system *E. coli* BL21/pET28a-*lipAB* and two dual expression plasmid systems *E. coli* BL21/pETDuet-*lipA*-*lipB* and *E. coli* BL21/pACYCDuet-*lipA*-*lipB* were successfully constructed. The lipase activities of the three expression systems were compared to choose the optimal expression method. Under the same cultured condition, the activities of the lipases expressed by *E. coli* BL21/pET28a-*lipAB* and *E. coli* BL21/pETDuet-*lipA*-*lipB* were 1300 U/L and 3200 U/L, respectively, while the activity of the lipase expressed by *E. coli* BL21/pACYCDuet-*lipA*-*lipB* was up to 8500 U/L. The lipase LipA had an optimal temperature of 30°C and an optimal pH of 9 with a strong pH tolerance. The active LipA could catalyze the reaction between fatty alcohols and fatty acids to generate fatty acid alkyl esters, which meant that LipA was able to catalyze esterification reaction. The most suitable fatty acid and alcohol substrates for esterification were octylic acid and hexanol, respectively.

**Conclusions:**

The effect of different plasmid system on the active LipA expression was significantly different. pACYCDuet-*lipA*-*lipB* was more suitable for the expression of active LipA than pET28a-*lipAB* and pETDuet-*lipA*-*lipB*. The LipA showed obvious esterification activity and thus had potential biocatalytic applications. The expression method reported here can give reference for the expression of those enzymes that require chaperones.

## Background

Lipases (triacylglycerol acylhydrolases, EC 3.1.1.3) catalyze the hydrolysis and synthesis of a variety of acylglycerols at the interface of lipid and water [1]. Owing to the properties like wide substrate specificity, high enantio- and regioselectivity, lipases have a wide range of potential applications in industry such as organic synthesis, detergent formulation, food and pharmaceutical industries [2,3]. *Pseudomonas* lipases display special biochemical characteristics which are not common among the lipases produced by other microorganism. For example, it has exhibited amide hydrolyzing activity [4] and high enantioselectivity towards hydrolysis of racemic esters [5,6].

In spite of various potential applications of *Pseudomonas* lipases, their functional overexpression requires a lipase-specific chaperone to fold into an active conformation [7,8]. Although the lipases can be produced by their homologous expression in *Pseudomonas* hosts, many *Pseudomonas* strains are potential pathogens and special safety regulations need to govern their cultivation. Therefore, it is necessary to set up a heterologous expression system and develop efficient refolding procedure.

Recent years, many microbial lipase genes had been isolated, cloned, sequenced and overexpressed in homologous or heterologous hosts such as *Escherichia coli*, filamentous fungi or yeast. The expression of recombinant proteins in bacteria hosts often results in the formation of inclusion bodies which are insoluble and inactive. Many methods had been used to obtain high active expression amount of *Pseudomonas* lipases [9-12]. The amount of active enzyme achieved by co-expression of *Pseudomonas* lipase and its cognate foldase in an expression vector was low because of the complexity of the gene regulatory and secretion mechanisms [13-15]. Therefore, two expression vectors had been used to solve this problem. For example, Madan B and Mishra P [16] put the *lipA* and *lipB* under the influence of strong T7 promoter in pET32Xa/LIC and pET29a vectors, respectively. Then *E. coli* BL21 (DE3) containing pET*lipA* and pET*lipB* was expressed upon induction with IPTG, and the productivity attained to 812 U L^-1^ h^-1^. Akbari et al. [17] also subcloned the lipase and lipase specific foldase into two separate expression vectors and expressed in *E. coli* as inactive inclusion bodies and soluble form, respectively. Then the lipase and lipase special foldase were mixed *in vitro* to refold active lipase. The effect of different concentrations of various additives on the lipase refolding was investigated, and the best yield of 70 IU/ml was obtained.

In this work, a lipase-producing strain was screened from oil-rich soil and identified as *Pseudomonas aeruginosa* through 16S rDNA sequence analysis. Then the lipase gene *lipA* and the lipase special foldase gene *lipB* were successfully cloned and sequenced. The expression vectors pACYCDuet-1 and pETDuet-1 containing two multiple cloning sites (MCS) were used to co-express the two target genes. *In vivo* expression of the active lipase was successfully achieved. To date, only one report by Quyen et al. [18] achieved the expression of *in vivo* functional lipase in heterologous host *E. coli* using a dual expression plasmid system which was derived from pET22b. It demonstrated that a dual expression plasmid system *E. coli* could overproduce and secrete the active chaperone-dependent lipase (subfamilies I.1 and I.2) *in vivo*. An improved dual expression plasmid system *E. coli* could be potentially applied for industrial-scale production of subfamily I.1 and I.2 lipases.

## Results and discussion

### Identification of the lipase-producing strain

**Figure 1:**
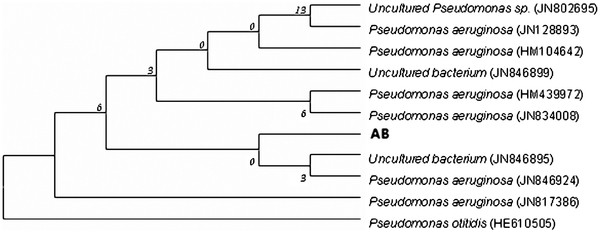
**Phylogenetic analysis by 16S rRNA gene sequencing of strain AB.** Phylogenetic analysis was performed using the program MEGA4.0. The length of each branch pair represents the evolutionary distance between the sequences.

Initially, one isolate named AB from oil-rich samples enriched on Rhodamine B agar plates was selected through the screening procedure. For precise identification, 16S rRNA gene sequencing of the isolate was performed and nucleotide sequence generated was aligned and analyzed for identification of bacterial species. Through the phylogenetic tree analysis, 16S rRNA gene sequence of strain AB was compared with the sequences of ten *Pseudomonas* species. This strain had a high degree of homology (99% 16S rRNA gene sequence similarity) with *Pseudomonas aeruginosa* (Figure 1), and thus was identified as *Psedomonas aeruginosa*.

### Cloning and sequence analysis of lipase and lipase foldase genes from strain AB

Genomic DNA obtained from the strain AB was used to isolate the lipase and lipase specific foldase genes. The lipase gene named *lipA* consisted of 936 nucleotides, encoding 311 amino acids, and the size of lipase specific foldase gene named *lipB* was 1023 bp, encoding 340 amino acids. Both genes had 99% homology with reported sequences that from *Pseudomonas aeruginosa* PAO1 (AE004091.2) or *Pseudomonas aeruginosa* lip9 (AB290342.1). The gene *lipA* had nine nucleotides different from that of *Pseudomonas aeruginosa* PAO1, but only one amino acid was different (Lys was replaced by Arg). The gene *lipB* which encoded lipase-specific chaperone was coexpressed along with the structure gene of *lipA* in the same operon in *P.aeruginosa*. The gene *lipAB* which contained both *lipA* and *lipB* consisted of 2008 nucleotides, and there were 49 bp between *lipA* and *lipB*. The nucleotide sequences of *lipA* (936 bp) and *lipB* (1023 bp) had been deposited in the GenBank database under accession numbers JN594060 and JN594061, respectively.

### Expression of the lipase using different expression plasmid systems

In this work, three expression plasmids, one single expression plasmid pET28a and two dual expression plasmids pACYCDuet-1 and pETDuet-1, were investigated for high level expression of the active lipase. pACYCDuet-1 and pETDuet-1 are both designed for the coexpression of two target genes. Each of the vectors contains two multiple cloning sites (MCS), and each of which is preceded by a T7 promoter/*lac* operator and ribosome binding site (rbs) [19]. The vector pACYCDuet-1 carries the P15A replicon, *lacI* gene and chloramphenicol resistance gene. While the vector pETDuet-1 carries the pBR322-derived ColE1 replicon, *lacI* gene and ampicillin resistance gene. pACYCDuet-1 and pETDuet-1 can be used in combination in an appropriate host strain for the coexpression of up to 4 target genes.

**Figure 2:**
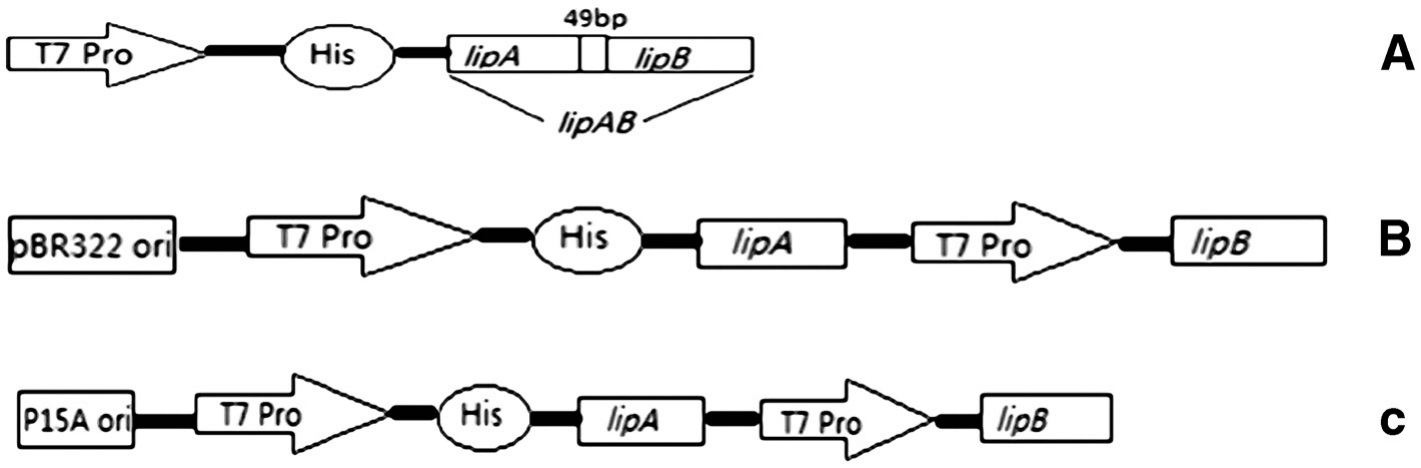
**Schematic representation of the expression cassettes.** (**A**) pET28a-*lipAB*; (**B**) pETDuet-*lipA*-*lipB*; (**C**) pACYCDuet-*lipA*-*lipB*. T7 Pro, T7 promoter; His, 6 × His tag; P15A ori, P15A origin; pBR322 ori, pBR322 origion; *lipA*, mature lipase gene; *lipB*, the complete chaperone gene; *lipAB*, the gene contained both *lipA* and *lipB*.

Three different expression plasmids pACYCDuet-*lipA*-*lipB*, pETDuet-*lipA*-*lipB* and pET28a-*lipAB* contained *lipA*, *lipB* or *lipAB* genes were successfully constructed. The structure schematic diagram was shown in the Figure 2.

**Figure 3:**
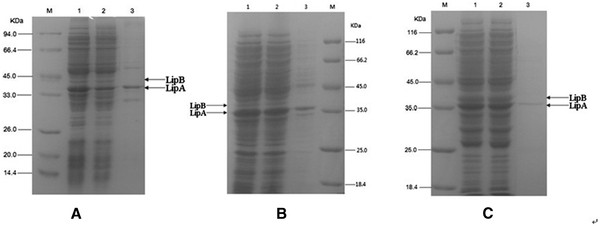
**SDS-PAGE gel stained with Coomassie brilliant blue R-250 showing expression of recombinant proteins.** (**A**) M, Protein molecular weight marker. Lane 1, pET28a-*lipAB* whole cell lysate. Lane 2, cell lysate's supernatant. Lane 3, cell lysate's precipitate; (**B**) M, Protein molecular weight marker. Lane 1, pETDuet-*lipA*-*lipB* whole cell lysate. Lane 2, cell lysate's supernatant. Lane 3, cell lysate's precipitate; (**C**) M, Protein molecular weight marker. Lane 1, pACYCDuet-*lipA*-*lipB* whole cell lysate. Lane 2, cell lysate's supernatant. Lane 3, cell lysate's precipitate.

The three recombinant plasmids were transferred into *E.coli* BL21 (DE3) and expressed under the control of T7 promoter, respectively. Upon induction with 0.5 mM IPTG for 20 h at 20°C, the cells were collected by centrifugation and disrupted by sonication. Both polypeptides LipA (35 KDa) and LipB (38 KDa) were expressed as shown in SDS-PAGE gel (Figure 3).

The whole cell expression level of LipA was almost the same in the three expression plasmid systems *E. coli* BL21/pET28a-*lipAB* (Figure 3A, lane1), *E. coli* BL21/pETDuet-*lipAlipB* (Figure 3B, lane 1) and *E. coli* BL21/pACYCDuet-*lipAlipB* (Figure 3C, lane 1) in the same condition. However, the active LipA level in the whole cell lysate's supernatant was obviously different among the three expression plasmid systems. The amount of active LipA expressed by the *E. coli* BL21/pET28a-*lipAB* (Figure 3A, lane2) and the *E. coli* BL21/pETDuet-*lipAlipB* (Figure 3B, lane 2) was less than that of the *E. coli* BL21/pACYCDuet-*lipAlipB* (Figure 3C, lane 2). The expression level of LipB was also significantly different among the three expression plasmid systems. The amount of the LipB expressed by the *E. coli* BL21/pACYCDuet-*lipAlipB* (Figure 3C) was much more than those of the other two expression plasmid systems. The lipase activity of different recombinant *E. coli* cell lysate's supernatants containing active lipase was also determined. The result showed that the activity of plasmid pET28a-*lipAB* system was only 1300 U/L, and the activity of plasmid pETDuet-*lipAlipB* system was 3200 U/L, but that of plasmid pACYCDuet-*lipAlipB* system could up to 8500 U/L, which was more than six times than the single expression plasmid pET28a-*lipAB* system. These results indicated that LipB was essential for the production of active LipA, which was agreement with many other reports. Gerritse et al. [20] found that the expression level of the LipB played a key role in the expression and secretion of the active lipase when the copy number of the plasmid carrying the exogenous gene was more than 10, and only when the LipB expression amount was raised, the activity of the lipase could be improved. Also, Quyen et al. [21] reported that the active lipase was negligible when the coexpressed chaperone LipB in the gene cluster was expressed at a negligible level in comparison to the LipA.

The ratio of lipase and chaperone was important to the lipase activity. Traub et al. [22] reported that the lipase and chaperone must be present in equimolar amounts to obtain optimal refolding efficiency. Akbari et al. [17] also reported that the concentration of lipase and foldase was an important parameter affecting refolding process. In this work, the SDS-PAGE showed that the concentration of LipA and LipB was in a more equal proportion in pACYCDuet-*lipAlipB* system than those of pETDuet-*lipAlipB* system and pET28a-*lipAB* system (Figure 3A-C). Therefore, the LipA expressed by pACYCDuet-*lipAlipB* system could be more refolded into active conformation. This can explain the result that the lipase activity expressed by pACYCDuet-*lipAlipB* system was more than those of pETDuet-*lipAlipB* system and pET28a-*lipAB* system in the same condition.

In addition, the two dual expression plasmids pACYCDuet-1 and pETDuet-1 have different replicons, so they can be recombined in the same host strain for the overexpression of target genes. In this work, the two plasmids pACYCDuet-*lipA*-*lipB* and pETDuet-*lipA*-*lipB* were transferred into the same *E. coli* BL21 to hope further improvement of the LipA and LipB expression. After induction with IPTG, the activity of the lipase expressed by *E. coli* BL21/pACYCDuet-*lipA*-*lipB +* pETDuet-*lipA*-*lipB* was only 3000 U/L, which was lower than that of *E. coli* BL21/pETDuet-*lipA*-*lipB* or *E. coli* BL21/pACYCDuet-*lipA*-*lipB* single plasmid system. The copy number of pACYCDuet-*lipA*-*lipB* or/and pETDuet-*lipA*-*lipB* may decrease when the two plasmids existed in the same host strain.

As previously reported, the gene regulatory and secretion mechanisms were complex by coexpression of lipase and its special foldase in an expression vector. In this work, two dual expression plasmids systems were used to enhance the expression amount of the LipB in *E. coli* in order to obtain more active lipase *in vivo*. The result showed that the dual expression plasmids could obviously increase the expression level of active lipase. But different dual expression plasmid showed different effect, and the plasmid pACYCDuet-1 was more suitable for the co-expression LipA and LipB than the plasmid pETDuet-1. So far, only Quyen et al. [18] had chose a dual expression plasmid system to express an active lipase, but the dual expression plasmid was derived from pET22b and its construction process was complex. The dual expression plasmids pACYCDuet-1 and pETDuet-1 were both the commercial plasmids, which were more convenient for practical application.

### Characterization of the lipase

To examine the properties of the lipase produced by *E. coli* BL21/pACYCDuet-*lipA*-*lipB*, the recombinant lipase was purified to homogeneity (more than 95%) using nickel affinity chromatography (Ni-NTA) by exploiting the histidine tag.

**Figure 4:**
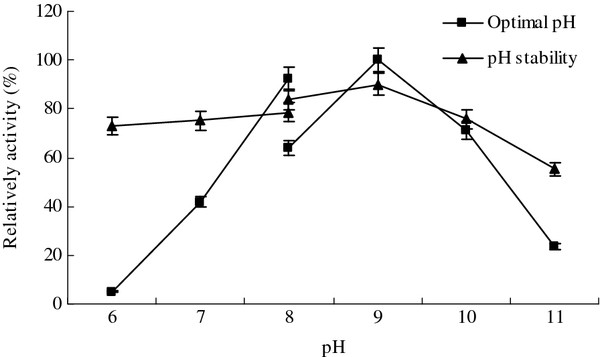
**Effect of the pH on the lipase activity and stability.** The highest lipase activity was 129 U/ml and set to 100%.

The optimum pH of the lipase was found to be 9.0, and the enzyme activity was also relatively high at the pH range of 8–10 (Figure 4). The lipase showed good pH stability, and more than 70% of the original activity could be retained at the pH range of 6–10 (Figure 4). The optimum pH and pH stability revealed that the lipase should be alkaline in nature. Lipases from other *Pseudomonas* sp. such as *P. mendocina* PK-12CS [23] and *P. aeruginosa*[24] had also been reported to possess good stability over wide pH range.

**Figure 5:**
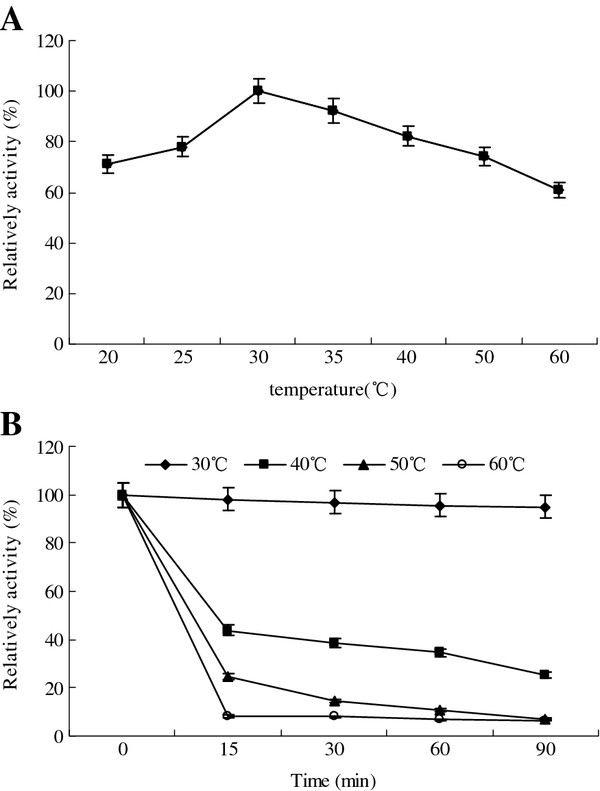
**Effect of the temperature on the lipase activity (A) and stability (B).** The highest lipase activity was 164 U/ml and set to 100%.

The optimal temperature of the lipase was observed to be 30°C (Figure 5A), which showed that this lipase was a low temperature enzyme. The thermal stability of the lipase was poor. Although the enzyme activity had almost no change with the extension of time at 30°C, it declined intensively at 40°C, and there was only less than 40% of the original activity retained at 90 min. When the temperature was above 50°C, the enzyme activity lost seriously only after 15 min (Figure 5B).

**Figure 6:**
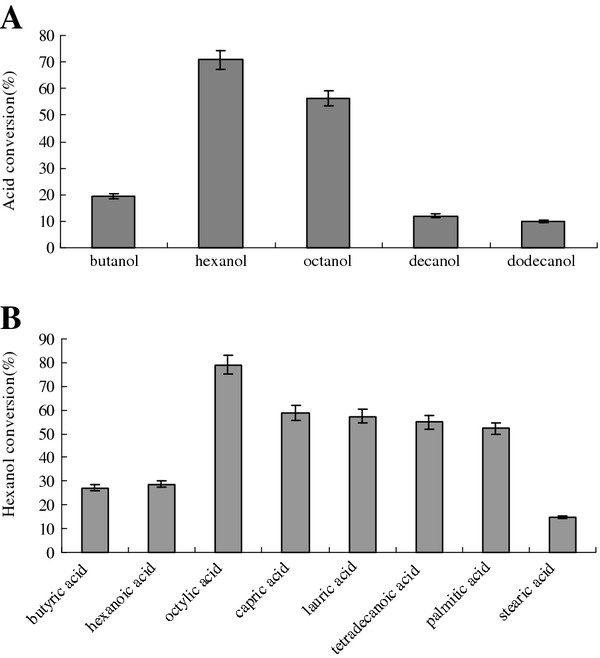
**The optimum substrate alcohol and fatty acid for lipase catalyzed esterification reaction.** (**A**) The optimal alcohol; (**B**) The optimal fatty acid.

The lyophilized lipase catalyzed the esterification reaction between butyric acid and hexanol was first investigated. An obvious peak appeared through GC analysis, and was identified as butyric acid hexyl ester. This indicated that the lipase could catalyze the esterification reaction. The optimum substrate alcohol and fatty acid for this lipase catalyzed esterification reaction were further investigated. The result was shown in Figure 6. Medium chain alcohols were appropriate with hexanol as the optimal alcohol (Figure 6A). The optimal fatty acid was octylic acid, and the 10 to 16 carbon acid was also fine, which meant that the lipase was suitable to catalyze the esterification of long chain fatty acids (Figure 6B).

## Conclusions

In this work, a lipase-producing strain identified as *P. aeruginosa* was isolated through the screening procedure. The lipase gene *lipA* (936 bp) and lipase specific foldase gene *lipB* (1023 bp) were cloned from the *P.aeruginosa*. Three heterologous expression systems were established for the expression of active lipase *in vivo*. The plasmid pACYCDuet-*lipA*-*lipB* was the optimal expression plasmid among the three expression plasmids constructed in this work, and the lipase activity produced by *E. coli* BL21/pACYCDuet-*lipA*-*lipB* could be up to 8500 U/L. The lipase had an optimal temperature of 30°C and an optimal pH of 9. The lipase showed obvious esterification activity and thus had potential biocatalytic applications.

## Methods

### Materials

Restriction enzymes, T4 DNA ligase, and Taq polymerase premix were supplied by MBI Fermentas (Germany). The DNA gel extraction and plasmid extraction were purchased from Generay Biotech (Shanghai) Co., Ltd. Peptone and yeast extract were obtained from Oxoid. All reagents were of analytical grade unless otherwise stated.

### Strains, plasmids and cultured conditions

Soil samples were collected from oil-rich soil near restaurants or residential area. *E. coli* DH5α was used for transforming and cloning recombinant plasmids, and *E. coli* BL21 (DE3) was used as a host for gene expression. Plasmids pET28a, pACYCDuet-1 and pETDuet-1 preserved in our laboratory were used for the construction of the expression systems. The antibiotic concentrations used for the optimum growth of strains were 100 μg/ml of ampicillin, 50 μg/ml of kanamycin and 100 μg/ml of chloramphenicol. *E. coli* was cultured at 37°C in Luria-Bertani medium (LB) supplemented with antibiotics. Protein expression was induced by the addition of 0.5 mM IPTG to the growth medium.

### Isolation of lipase-producing strain

LB medium added with 1% olive oil emulsion was used as enrichment medium. The culture was repeatedly transferred to fresh medium for three times. Then, samples of the culture were diluted and spread on Rhodamine B agar plates (yeast extract 0.5%, tryptone 1%, NaCl 1%, olive oil 1%, Rhodamine B 0.001% and agar 1.5%). The plates were incubated at 37°C for 48 h. The microbes showing obviously red hydrolysis circle were pointed on the new Rhodamine B agar plates. The ones showing high ratios of red hydrolysis circle were selected as potential lipase producers and identified by means of 16S rDNA.

### DNA manipulations

Chromosomal DNA of the screened strain was obtained from cells grown in LB medium at 200 rpm at 37°C for 24 h as described by Ausubel et al. [25] with some modification, as the precipitation of DNA was carried out using ethanol instead of isopropanol. Plasmid DNA was isolated by alkaline lysis method [26]. The oligonucleotide primers used in this study were synthesized by Generay Biotech (Shanghai) Co., Ltd.

### Cloning of lipase and lipase specific foldase genes

The genomic DNA from the screened *P. aeruginosa* AB was used as a source of lipase and lipase specific foldase genes. PCR primers (Table 1) for the lipase and lipase specific foldase genes were designed based on the full-length DNA sequences reported for *P. aeruginosa* lipase and foldase genes in NCBI GenBank.

**Table 1:** Primers used in this work (F: forward primer, R: reverse primer)

**Primers**	**Sequence (5’-3’)**	**Source**
A-F	GGATCCATGAAGAAGAAGTCTCTGCT	Amplification of *lipA*, *Bam*HI site
A-R	AAGCTTCTACAGGCTGGCGTTCTT	Amplification of *lipA*, *Hin*dIII site
B-F	GGGCATATGGTGAAGAAAATCCTCCTG	Amplification of *lipB*, *Nde*I site
B-R	CTCGAGTCAGCGCTGCTCGGCCT	Amplification of *lipB*, *Xho*I site
AB-F	CCTTGGATCCATGAAGAAGAAGTCTCTGCTCC	Amplification of *lipAB*, *Bam*HI site
AB-R	CCTTAAGCTTCAGCTGCTCGGCCTGGCGCAT	Amplification of *lipAB*, *Hin*dIII site

For cloning the lipase gene *lipA* and lipase specific foldase gene *lipB*, following oligonucleotides in the Table 1 were used for PCR amplification. Each reaction mixture (50 μl) contained premix 25 μl, ddH_2_O 22 μl, sense primer (20 μM) 1 μl, antisense primer (20 μM) 1 μl and genomic DNA 1 μl. While the gene named *lipAB* contained both *lipA* and *lipB* had a higher G + C content than the usual gene, 2 μl DMSO was added to the reaction mixture. The PCR for *lipA* consisted of an initial denaturation for 5 min at 94°C. Thirty cycles were run, and each cycle consisted of 1 min of denaturation at 94°C, 45 s of annealing at 60°C and 1 min of extension at 72°C followed by a final extension of 10 min at 72°C. For amplification of *lipB*, annealing temperature was 53°C. For amplification of the gene *lipAB* contained both *lipA* and *lipB*, step down PCR programme was run, which consisted of six steps with annealing temperature declined from 65°C to 55°C at the interval of 2°C. All of the resultant PCR products were then ligated with plasmid pMD19-T, respectively. The constructed plasmids T-*lipA*, T-*lipB* and T-*lipAB* were sequenced by BGI and blasted on NCBI.

### Construction of the expression plasmids

The constructed plasmid T-*lipA* which contained *lipA* gene was digested with *Bam*HI and *Hin*dIII and ligated into similarly digested pACYCDuet-1 and pETDuet-1 vectors using T4 DNA ligase, respectively. The re-constructed plasmids were named as pACYCDuet-*lipA* and pETDuet-*lipA*, respectively. Similarly, the constructed plasmid T-*lipB* which contained *lipB* gene was digested with *Nde*I and *Xho*I and ligated into similarly digested pACYCDuet-*lipA* and pETDuet-*lipB* vectors using T4 DNA ligase, respectively. The re-constructed plasmids were named as pACYCDuet-*lipA*-*lipB* and pETDuet-*lipA*-*lipB*, respectively (see the Additional file 1 (B) and (C) genetic maps). The constructed plasmid T-*lipAB* which contained both *lipA* and *lipB* was digested with *Bam*HI and *Hin*dIII and ligated into similarly digested pET28a. The re-constructed plasmid was named as pET28a-*lipAB* (see the Additional file 1 (A) genetic map). The construction process of the recombinant plasmid pACYCDuet-*lipA*-*lipB* was shown in the Additional file 2. The other two recombinant plasmids were also constructed through the similarly process.

Competent *E. coli* DH5α cells prepared by CaCl_2_ method were transformed using the heat shock and cold treatment [25]. All the transformed colonies were screened by colony PCR followed by double digestion of the constructed plasmid.

### Functional expression of recombinant lipase in *E. coli*

All the recombinant plasmids were transformed into *E. coli* BL21 (DE3) for protein overexpression. The transformants were grown in LB medium added with the corresponding antibiotic at 37°C and 200 rpm. When the optical density (OD) at 600 nm reached about 0.8, IPTG was added to a final concentration of 0.5 mM. Incubation at 20°C for a further 20 h, cells were harvested by centrifugation (8,000 rpm, 5 min at 4°C). The pellet was washed with 50 mM Tris–HCl buffer (pH 8.0) and was resuspended in 10 ml of the same buffer. The cells were then disrupted by sonication at the power controlled from 200 W to 400 W, and each circle worked 4 s and intermitted 5 s, continued 20 min. Bacterium fluid should be held in ice bath during the sonication to prevent protein denaturation. The cell lysate was centrifuged at 10,000 rpm for 10 min. The supernatant was collected as soluble fraction, while the pellet was taken as the insoluble fraction and resuspended in an equal volume of Tris–HCl buffer (pH 8.0).

In order to detect the recombinant protein, 20 μl of samples were analyzed by sodium dodecyl sulfate (SDS) gel electrophoresis (GE) as described by Laemmli [27]. Proteins were stained with Coomassie Brilliant Blue R-250. The soluble fraction which contained active lipase was subjected to activity determination by alkali titration method.

### Determination of the lipase activity

Two methods were adopted to determine the lipase activity in this work. One method estimated the free fatty acid by alkali titration using olive oil as the substrate [28]. 1 ml enzyme solution was added to the substrate solution containing 5 ml of 10% emulsified olive oil in 10% gum acacia and 2.5 ml of 0.2 mol/L Tris–HCl buffer (pH 8.0). The enzyme-substrate solution was incubated on water bath shaker at 30°C and 150 rpm for 10 min. 10 ml ethanol was added to stop the reaction. Liberated fatty acids were titrated with 0.05 mol/L NaOH using phenolphthalein as indicator. One unit (U) was defined as the amount of enzyme that released 1 μmol fatty acid per minute under the standard assay conditions. This method was used to determine the expressed lipase activity of different recombinant *E. coli.*

The other method determined the liberated *p*NP using *para*-nitrophenyl palmitate (*p*NPP) as substrate [29] with slight modifications. The *p*NPP was dissolved in 2-propanol to make the concentration of 3 mg/ml. The substrate solution consisted of 0.1 ml of *p*NPP solution and 2.8 ml Tris–HCl buffer (20 mM, pH8). The reaction was carried out at 30°C by adding 0.1 ml appropriately diluted enzyme solution to the substrate solution preincubated at 30°C for 5 min, and incubation was continued for further 5 min. The test tubes were immersed in ice till the optical density was taken at 405 nm. One unit (U) was defined as the amount of enzyme that liberated 1 μmol *p*NP per minute under the standard assay conditions. This method was used to assay the characterization of lipase.

### Characterization of lipase

The optimum pH for the purified lipase was evaluated over a pH range from 6.0 to 11.0 under the standard assay procedures. 20 mM of sodium phosphate buffer (pH6.0-8.0) and borax buffer (8.0-11.0) were used. The pH stability of the lipase was investigated at 30°C by preincubation of the enzyme solutions in the described buffer systems in the absence of substrate for 30 min. The reaction mixture was then subjected to the standard assay procedures and a pH profile was produced with the enzyme activity at the optimum pH set to 100%.

The optimum temperature for the purified lipase was measured at various temperatures (20-50°C) under standard assay procedures. A temperature profile was produced with the enzyme activity at the optimum temperature set to 100%. The thermostability of the lipase was investigated by preincubating the enzyme solutions at various temperatures (20-50°C) for 15, 30, 60 and 90 min, respectively. The residual lipase activity of the samples was then assayed under standard assay procedures.

In order to investigate the esterification characterization of the lipase, the purified lipase was lyophilized for 12 h, and the dried powder was used as the lipase catalyst. The reaction mixture contained 1 mmol alcohol, 1 mmol fatty acid and 5 ml *n*-hexane. 5.0 mg lipase powder was added into the reaction mixture and the reaction was carried out at 30°C for 8 h. 1 ml of reaction liquid was centrifuged at 8000 rpm for 5 min, and the supernatant was for gas chromatography (GC) analysis. The product was identified by GC-MS. Butanol, hexanol, octanol, decanol and dodecanol were selected to esterify with hexanoic acid for optimum alcohol substrate investigation. Butyric acid, hexanoic acid, octylic acid, capric acid, lauric acid, tetradecanoic acid, palmitic acid and stearic acid were selected to esterify with hexanol for optimum fatty acid substrate investigation. GC analysis was performed on an Agilent 6890 Gas Chromatograph equipped with a flame ionization detector (FID) and a HP-5 capillary column (5% phenyl methyl siloxane capillary, 30.0 m × 320 μm × 0.25 μm nomimal). The column temperature was kept at 60°C for 0.5 min, heated to 220°C at 10°C/min. The temperatures of the injector and detector were set at 250°C.

## Competing interests

The authors declare that they have no competing interests.

## Authors' contributions

XPW and JJX performed the cloning and expression of lipase. XPW and EZS drafted the manuscript. PYY performed the characterization of lipase and revision of the manuscript. BG commented on and polished the manuscript. EZS and DZW supervised the study. All authors read and approved the final manuscript.

## Supplementary Material

Additional file 1 **Genetic maps of recombinant plasmids pET28a-*lipAB*(A), pETDuet-*lipA*-*lipB*(B) and pACYCDuet-*lipA*-*lipB*(C).** Click here for file

Additional file 2 **The schematic of pACYCDuet-*lipA*-*lipB*construction process.** The constructed plasmids T-*lipA* which contained *lipA* gene was digested with BamHI and HindIII and ligated into similarly digested pACYCDuet-1 vector using T4 DNA ligase. The re-constructed plasmid pACYCDuet-*lipA* and the constructed plasmid T-*lipB* which contained *lipB* gene was digested with NdeI and XhoI, repectively. Then the two digested vectors were ligated using T4 DNA ligase to get the recombinant plasmid pACYCDuet-*lipA*-*lipB*. Click here for file
