# Supplementary figures and images for: In vivo functional expression of a screened P. aeruginosa chaperone-dependent lipase in E. coli
Source: BMC Biotechnol. 2012 Sep 6;12:58. doi: 10.1186/1472-6750-12-58 (PMC3497882; doi:10.1186/1472-6750-12-58)

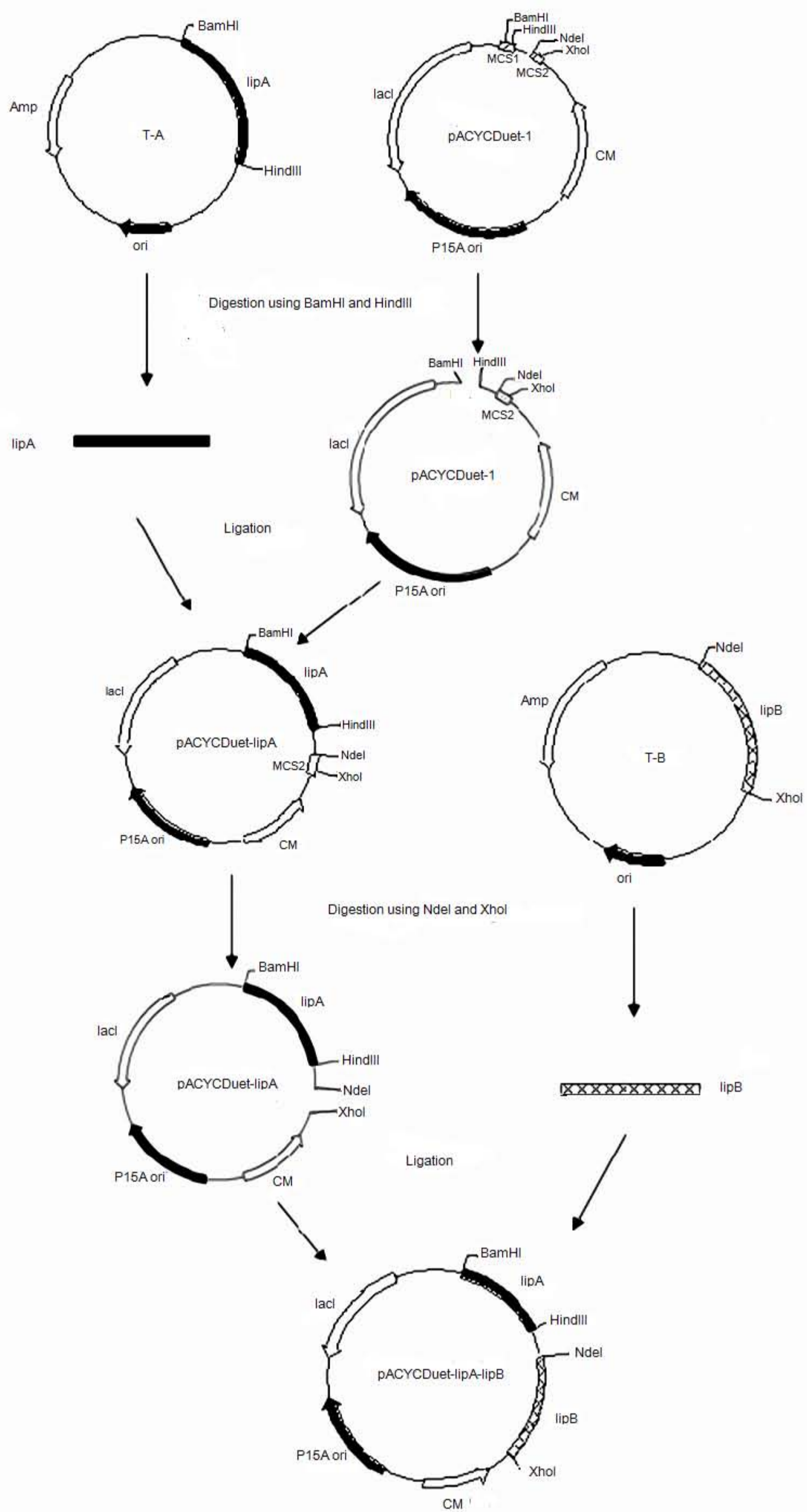

Supplement: Additional file 2 — The schematic of pACYCDuet-lipA-lipBconstruction process. The constructed plasmids T-lipA which contained lipA gene was digested with BamHI and HindIII and ligated into similarly digested pACYCDuet-1 vector using T4 DNA ligase. The re-constructed plasmid pACYCDuet-lipA and the constructed plasmid T-lipB which contained lipB gene was digested with NdeI and XhoI, repectively. Then the two digested vectors were ligated using T4 DNA ligase to get the recombinant plasmid pACYCDuet-lipA-lipB. [file 1472-6750-12-58-S2.pdf]
