# Supplementary material for: In vivo functional expression of a screened P. aeruginosa chaperone-dependent lipase in E. coli
Source: BMC Biotechnol. 2012 Sep 6;12:58. doi: 10.1186/1472-6750-12-58 (PMC3497882; doi:10.1186/1472-6750-12-58)
Supplement: Additional file 1 — Genetic maps of recombinant plasmids pET28a-lipAB(A), pETDuet-lipA-lipB(B) and pACYCDuet-lipA-lipB(C). [file 1472-6750-12-58-S1.pdf]

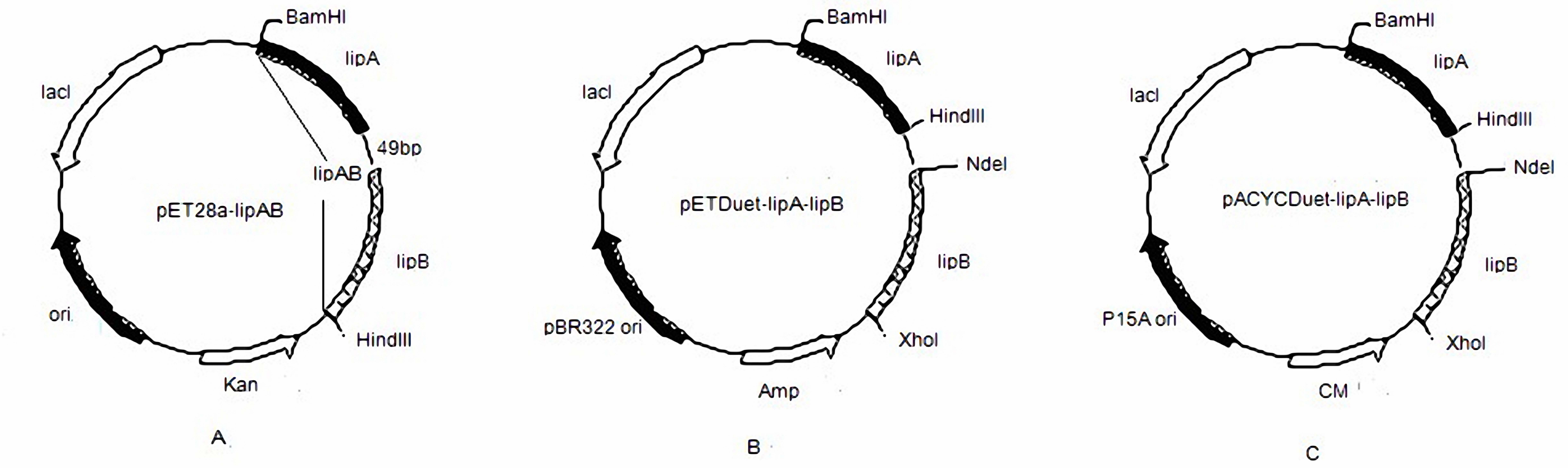

BamHI
lipA
lacI
49bp
lipAB
pET28a-lipAB
lipB
ori
HindIII
Kan
A
BamHI
lipA
lacI
HindIII
NdeI
pETDuet-lipA-lipB
lipB
pBR322 ori
XhoI
Amp
B
BamHI
lipA
lacI
HindIII
NdeI
pACYCDuet-lipA-lipB
lipB
P15A ori
XhoI
CM
C
